# Regulation of serotonin production by specific microbes from piglet gut

**DOI:** 10.1186/s40104-023-00903-7

**Published:** 2023-08-05

**Authors:** Ziyu Liu, Yidan Ling, Yu Peng, Shuibing Han, Yuting Ren, Yujia Jing, Wenlu Fan, Yong Su, Chunlong Mu, Weiyun Zhu

**Affiliations:** 1 Laboratory of Gastrointestinal Microbiology, College of Animal Science and Technology, Nanjing Agricultural University, Nanjing, China; 2 National Center for International Research on Animal Gut Nutrition, National Experimental Teaching Demonstration Center of Animal Science, Nanjing Agricultural University, Nanjing, China; 3 Hubei CAT Biological Technology Co., Ltd., Wuhan, China; 4 Department of Biochemistry and Molecular Biology, Cumming School of Medicine, University of Calgary, Calgary, Canada

**Keywords:** Colon, *Lactobacillus*, Microbial metabolites, Serotonin

## Abstract

**Background:**

Serotonin is an important signaling molecule that regulates secretory and sensory functions in the gut. Gut microbiota has been demonstrated to affect serotonin synthesis in rodent models. However, how gut microbes regulate intestinal serotonin production in piglets remains vague. To investigate the relationship between microbiota and serotonin specifically in the colon, microbial composition and serotonin concentration were analyzed in ileum-cannulated piglets subjected to antibiotic infusion from the ileum when comparing with saline infusion. Microbes that correlated positively with serotonin production were isolated from piglet colon and were further used to investigate the regulation mechanisms on serotonin production in IPEC-J2 and a putative enterochromaffin cell line RIN-14B cells.

**Results:**

Antibiotic infusion increased quantities of *Lactobacillus amylovorus* (LA) that positively correlated with increased serotonin concentrations in the colon, while no effects observed for *Limosilactobacillus reuteri* (LR). To understand how microbes regulate serotonin, representative strains of LA, LR, and *Streptococcus alactolyticus* (SA, enriched in feces from prior observation) were selected for cell culture studies. Compared to the control group, LA, LR and SA supernatants significantly up-regulated tryptophan hydroxylase 1 (TPH1) expression and promoted serotonin production in IPEC-J2 cells, while in RIN-14B cells only LA exerted similar action. To investigate potential mechanisms mediated by microbe-derived molecules, microbial metabolites including lactate, acetate, glutamine, and γ-aminobutyric acid were selected for cell treatment based on computational and metabolite profiling in bacterial supernatant. Among these metabolites, acetate upregulated the expression of free fatty acid receptor 3 and TPH1 while downregulated indoleamine 2,3-dioxygenase 1. Similar effects were also recapitulated when treating the cells with AR420626, an agonist targeting free fatty acid receptor 3.

**Conclusions:**

Overall, these results suggest that *Lactobacillus amylovorus* showed a positive correlation with serotonin production in the pig gut and exhibited a remarkable ability to regulate serotonin production in cell cultures. These findings provide evidence that microbial metabolites mediate the dialogue between microbes and host, which reveals a potential approach using microbial manipulation to regulate intestinal serotonin biosynthesis.

**Supplementary Information:**

The online version contains supplementary material available at 10.1186/s40104-023-00903-7.

## Background

Serotonin (5-Hydroxytryptamine, 5-HT) is an important signaling molecule involved in neuroendocrine and paracrine functions in the body [1] that affects gastrointestinal motility in pigs. A majority of 5-HT is produced in the gut through the tryptophan metabolism in the enterochromaffin cells (ECs) by the tryptophan hydroxylase 1 (TPH1) [2]. Tryptophan in mammals could also be degraded along the kynurenine pathway under the action of indoleamine 2,3-dioxygenase 1 (IDO1) [3, 4]. Active 5-HT enters the surrounding epithelial cells via serotonin reuptake transporter (SERT) and is degraded into 5-hydroxyindoleacetic acid by monoamine oxidase A (Maoa) [5]. Gut-derived 5-HT can act as an endocrine factor that is associated with gastrointestinal motility, nutrient absorption [6, 7], and hepatic blood flow [8, 9]. Therefore, 5-HT exerts a substantial impact on the gastrointestinal tract and beyond [7, 10].

Accumulating evidences have demonstrated that intestinal microorganisms can affect the synthesis of 5-HT by directly affecting TPH activity in human cells or rodent models [11, 12]. However, the relationship between 5-HT and microbes remains unclear in pigs. Germ-free mouse model and antibiotic intervention have been widely used in investigating the relationship between gut microbes and the host function [11, 13–15]. In the mouse model, gut microbiota plays an important role in regulating 5-HT biosynthesis in the gut. Notably, intestinal spore-forming microbes could promote 5-HT biosynthesis in mice and regulate gut motility and homeostasis [16], partly via microbial metabolites such as butyrate, cholate, p-aminobenzoate, and tyramine [16, 17]. Pig has a distinct microbiota composition from rodents [18, 19]. Our previous studies have shown that the changed microbiota composition after antibiotic intervention would lead to decreased 5-HT levels in the hypothalamus of the pig due to the reduced aromatic amino acid levels in the gut and blood circulation [20]. Conversely, an increase in intestinal aromatic amino acids after the corn starch infusion enables more tryptophan to be used for 5-HT synthesis in the hypothalamus in pigs [21]. Nevertheless, how specific microbes regulate the 5-HT biosynthesis in the gut of pigs remains unknown.

In this study, we aimed to evaluate the potential relationship between 5-HT and gut microbes in piglets. Compared to germ-free model, microbiota manipulation by broad-spectrum antibiotics is more feasible in ileum-cannulated pigs to investigate host-microbiota interaction. Employing the ileum-cannulated piglets with antibiotic intervention, we were able to analyze the correlation between microbes and 5-HT in vivo. The bacteria with significantly positive correlations with 5-HT concentration in vivo were selected for subsequent studies in vitro. Bacteria without a significant correlation with 5-HT concentration were also selected as comparisons. For in vitro studies, the intestinal porcine epithelial cell (IPEC-J2) was employed as a commonly used model of intestinal epithelial cells. RIN-14B was used as a model of enterochromaffin cells that are major cell population responsible for serotonin production. The effect of selected bacterial supernatant on serotonin production was investigated using IPEC-J2 and RIN-14B cells. These findings provide insights into microbial regulation of the serotonin pathway in piglets.

## Materials and methods

### Experimental design and sample collection

The animal experiment was conducted at the animal facility at Nanjing Agricultural University. All procedures were approved by the Animal Care and Use Committee of Nanjing Agricultural University, which conformed to the Regulations for the Administration of Affairs Concerning Experimental Animals of China (authorization number SYXK (Su) 2017–0027). A total of 10 male pigs (Duroc × Landrace × Large, 35 d, average body weight 10.00 ± 0.50 kg) were subjected to the basal diet formulated to meet the nutrient requirements according to the National Research Council (NRC, 2012) [22] standards for piglets in Table S1. All pigs were fitted with a simple T-cannula in the distal ileum according to previous methods [23]. Briefly, all piglets were maintained under a heating pad with 5% isoflurane (95% oxygen) used as the inhalation anesthetic agent to minimize the pig's suffering during the surgery. To avoid infections and recover better from ileal surgery, all pigs were hypodermically injected with ceftriaxone sodium and kept in a metabolic cage under a comfortable environment with temperature maintained at 26 ± 2 °C, and free access to water and basal diet for 10 d. On day 45 of age, pigs (12.08 ± 0.28 kg) were randomly allocated to control (Con) or antibiotics (Abx) groups. The Con groups (*n* = 5) were infused with 10 mL saline via T-cannula every morning lasting for 25 d while the Abx groups were infused with 10 mL with a mixture of antibiotics (ampicillin, 150 mg/kg/d; gentamicin, 4 mg/kg/d; and metronidazole, 30 mg/kg/d) which showed broadly antibacterial activity against gut microbiota in pigs [24]. After 25 d of the experiment, all pigs were euthanized. The experimental timeline was shown in the supplemental Fig. S1. Colonic digesta and tissue were collected and stored at −80 °C before further analyses.

### DNA extraction and quantitative real-time qPCR analysis

Total genomic DNA was extracted from 300 mg colonic digesta per sample by using the Bacterial Genomic DNA Extraction Kit (MALLBIO, Nanjing, China). Quantities of bacteria were determined by qPCR using specific primers (Table S2). To constuct the standard plasmid, the 16S rRNA amplicon per taxon was cloned into a pMD-18 T Vector System (TaKaRa, Kusatsu, Japan). The recombinant plasmid was transformed into *Escherichia coli*, and plasmid DNA was extracted from *Escherichia coli* using the Plasmid Mini Preparation Kit (MALLBIO, Nanjing, China). The qPCR was performed in triplicates on Applied Biosystems QuantStudio 5 PCR System (Thermo Fisher Scientific, Massachusetts, USA) by using ChamQ Universal SYBR qPCR Master Mix (Vazyme, Nanjing, China). The copy number of each taxon in digesta (copy/g) was quantified by a standard curve calculated by plasmid gradient dilution.

### Isolation, purification, and identification of bacterial strains

Fresh colonic digesta was obtained from healthy male pigs (Duroc × Landrace × Large, 50 days of age) and was added with a volume ratio of 1:9 sterilized PBS as the preliminary sample solution. The sample solution was then serially diluted to 1:10^5^, 1:10^6^, 1:10^7^, and 1:10^8^ as the second sample solution. The secondary sample solution was spread on MRS agar plates and cultured at 37 °C in an anaerobic culture tank. Pure and single colonies were picked and transferred to MRS liquid medium. These steps were repeated two to three times to ensure that the bacterial liquid is pure.

PCR was used to amplify the full 16S rRNA of the targeted strain by using specific primers (Table S2) with 2 × Taq Master Mix (Vazyme, Nanjing, China). The 16S rRNA sequences were obtained by sequencing (Tsingke Biotechnology Co., Ltd., Beijing, China) and then blasted against NCBI database to determine strains with high homology (> 99%). Sequences of targeted strain were further aligned by clustalW in MEGA 11 (Molecular Evolutionary Genetic Analysis) for phylogenetic analysis using a neighbor-joining method. Bacterial characteristics were identified using a complete biochemical identification tube (Hopebiol, Qingdao, China) and Gram staining kit (Solarbio, Beijing, China).

### Bacterial growth curve and preparation of bacterial supernatant

To investigate the bacteria that may be related to serotonin production, we analyzed the correlation between intestinal bacteria and serotonin concentration from the in vivo study. Bacteria that showed significant correlation with serotonin and were available in the pure bacterial collection were selected for subsequent study. As per the analyses, *Lactobacillus amylovorus* was selected because of its significant positive correlation with 5-HT concentration in vivo. *Limosilactobacillus reuteri* was selected as a comparison against *L. amylovorus* because its weak correlation with 5-HT. *Streptococcus alactolyticus* was selected considering the increasing abundance in feces after antibiotic intervention in our previous study [20]. Our lab has maintained a representative strain of *L. amylovorus*, *L. amylovorus* S1, which was recovered to use for the current study.

Bacteria were all cultured in an MRS medium at 37 °C. Bacterial growth was evaluated by OD_600_ value in different growth stages using a microplate reader. The bacteria culture medium with OD_600_ = 1 was centrifuged at 4 °C and 12,000 × *g* for 10 min, and the supernatant was stored at −20 °C after passing a 0.22-μm filter membrane.

### Cell culture and cell viability analysis

IPEC-J2, which was a routinely maintained cell line in our lab, was cultured in DMEM/F 12 Medium (Procell, Wuhan, China) with 10% fetal bovine serum (FBS). The rat insulinoma RIN-14B cells (ATCC ID CRL-2059), were cultured in RPMI-1640 (Procell, Wuhan, China) with 10% FBS. Both cell lines were cultured at 37 °C in a mixture atmosphere of 5% carbon dioxide and 95% air.

For bacterial supernatant and metabolite treatments, cells were cultured without FBS when reached 80%–90% density on 12-well plates. The water-insoluble metabolites (kynurenine and kynurenic acid), GLPG0974 and AR420626 (MedChemExpress, New Jersey, USA) were dissolved in DMSO and then added to the medium, while the water-soluble metabolites (lactate, acetate, glutamine, GABA, succinate, propionate, butyrate, deoxycholate) were dissolved directly in the medium. After 24 h treatment, cell culture supernatant and remaining adherent cells were collected for further analyses.

Cell viability was measured by using a Cell Counting Kit 8 (CCK8) assay (MedChemExpress, New Jersey, USA) according to the manufacturer’s instructions.

### RNA extraction and real-time qPCR analysis

Total RNA was extracted from cells using an RNA Easy kit (Aidlab Bio Co., Ltd., Beijing, China). The RNA was transcribed into cDNA using HiScript III RT SuperMix for qPCR (Vazyme, Nanjing, China). Each sample was performed in triplicates on Applied Biosystems QuantStudio 5 PCR System (Thermo Fisher Scientific, Massachusetts, USA) by using ChamQ Universal SYBR qPCR Master Mix (Vazyme, Nanjing, China). Primer sequences were shown in Table S3. The mRNA expression of targeted genes was normalized to β-actin as the housekeeping gene and calculated by using the 2^−ΔΔCt^ method.

### Western blot analysis

Total proteins were extracted from RIN-14B cells with radioimmunoprecipitation protein lysate (Beyotime Technology, Shanghai, China) with a 1% protease inhibitor. Protein concentrations were measured and adjusted by the bicinchoninic acid protein assay kit (Beyotime Technology, Shanghai, China). Equal amounts of protein were separated by 12% SDS-PAGE and then transferred to polyvinylidene fluoride membranes (Millipore, Bedford, MA, USA). The following antibodies were diluted in TBST: 1:500 rabbit anti-TPH1 (BOSTER, Wuhan, China), 1:500 rabbit anti-IDO1 (Affinity Biosciences, Melbourne, USA), 1:1,000 rabbit anti-β-actin (Cell Signaling Technology, Boston, USA) and 1:5,000 goat anti-rabbit IgG-HRP (Cell Signaling Technology, Boston, USA). The intensity of protein products was visualized by chemiluminescence kits (Biosharp, Guangzhou, China) and detected in a chemiluminescence system (Tanon, Shanghai, China). The protein levels were quantified using ImageJ software and expressed as targeted protein/β-actin ratio.

### 5-HT measurement

The concentration of 5-HT was measured with an ELISA kit (MBE10189, MALLBIO, Nanjing, China), according to the manufacturer’s instructions.

Colon tissue was washed with cold PBS (0.01 mol/L, pH = 7.4) to remove residual blood, and the tissue was minced after weighing. The minced tissue was added with PBS at a volume ratio of 1:9 and adequate amount of 2 mm zirconium beads. To further lyse tissue cells, tissue samples were homogenized with a cell disrupter (Shanghai Jing Xin, China). The homogenate was centrifuged at 5,000 × *g* for 5 min, and the supernatant was collected for measurement.

The remaining adherent cells were digested using 0.25% trypsin solution with EDTA (Procell, Wuhan, China). After the cell suspension was centrifuged, the supernatant was removed and cells were re-suspended with PBS. The Hemocytometer was used to count cell numbers. The cell suspension was homogenized in tubes with zirconium beads using a cell disrupter. The solution was centrifuged at 5,000 × *g* for 5 min, and the supernatant was used for detection.

### Bacterial genome analysis

As mentioned before, representative strains of *L. amylovorus*, *L. reuteri*, and *S. alactolyticus* were selected for subsequent studies. The complete genome of *Lactobacillus amylovorus* S1 and *Limosilactobacillus reuteri* strain WHH1689 were downloaded from the NCBI genome database with PRJNA795261 and PRJNA431039. The *L. amylovorus* S1 was the same strain uploaded by our laboratory previously [25]. The *L. reuteri* strain WHH1689 showed the highest 16S rRNA sequence similarity with *L. reuteri* LGM among 33 existing *L. reuteri* genomes on NCBI (Fig. S2). The barrnap software was used to extract 16S rRNA from the bacterial genome sequence.

The complete genome of *Streptococcus alactolyticus* LGM was sequenced in Illumina Novaseq6000 for around 2 × 150 bp and PacBio Sequel II for around 20 kb at Biozeron Biotechnology Co., Ltd. (Shanghai, China). The AbySS (version 2.0.2, http://www.bcgsc.ca/platform/bioinfo/software/abyss) was used to perform genome assembly from Illumina short reads firstly. The Canu (version 1.8, https://github.com/marbl/canu) was used to assemble the PacBio corrected long reads secondly. The GapCloser (version 1.12, https://sourceforge.net/projects/soapdenovo2/files/GapCloser/) was used to fill up the remaining local inner gaps and correct the single base polymorphism for the final genome assembly. Gene prediction was performed using GeneMarkS (version 4.6b) and the genes of key enzyme that synthesized microbial metabolites were blasted against KEGG (http://www.genome.jp/kegg/) to perform functional annotation by blastp module.

### Measurement of metabolites in bacterial supernatant 

Concentrations of acetate and lactate in bacterial supernatant were measured with gas chromatography or high-performance liquid chromatography (Agilent 1220 Infinity LC system; Agilent Technologies, CA, USA), respectively, as described previously [25, 26]. Concentrations of gamma-aminobutyric acid (GABA) and glutamine were determined by ELISA kits (MALLBIO, Nanjing, China).

### Statistical analysis

All data were analyzed by using SPSS 20.0 (SPSS Inc., Chicago, USA) and visualized by using GraphPad Prism 9 (San Diego, CA, USA). The Student's *t*-test was used to detect significant differences between 2 groups while one-way ANOVA was used to detect significant differences for multiple comparisons with Fisher’s LSD test. The *P* value < 0.05 was considered statistically significant.

## Results

### Impact of hindgut antibiotic infusion on 5-HT levels and bacterial quantities

**Fig. 1:**
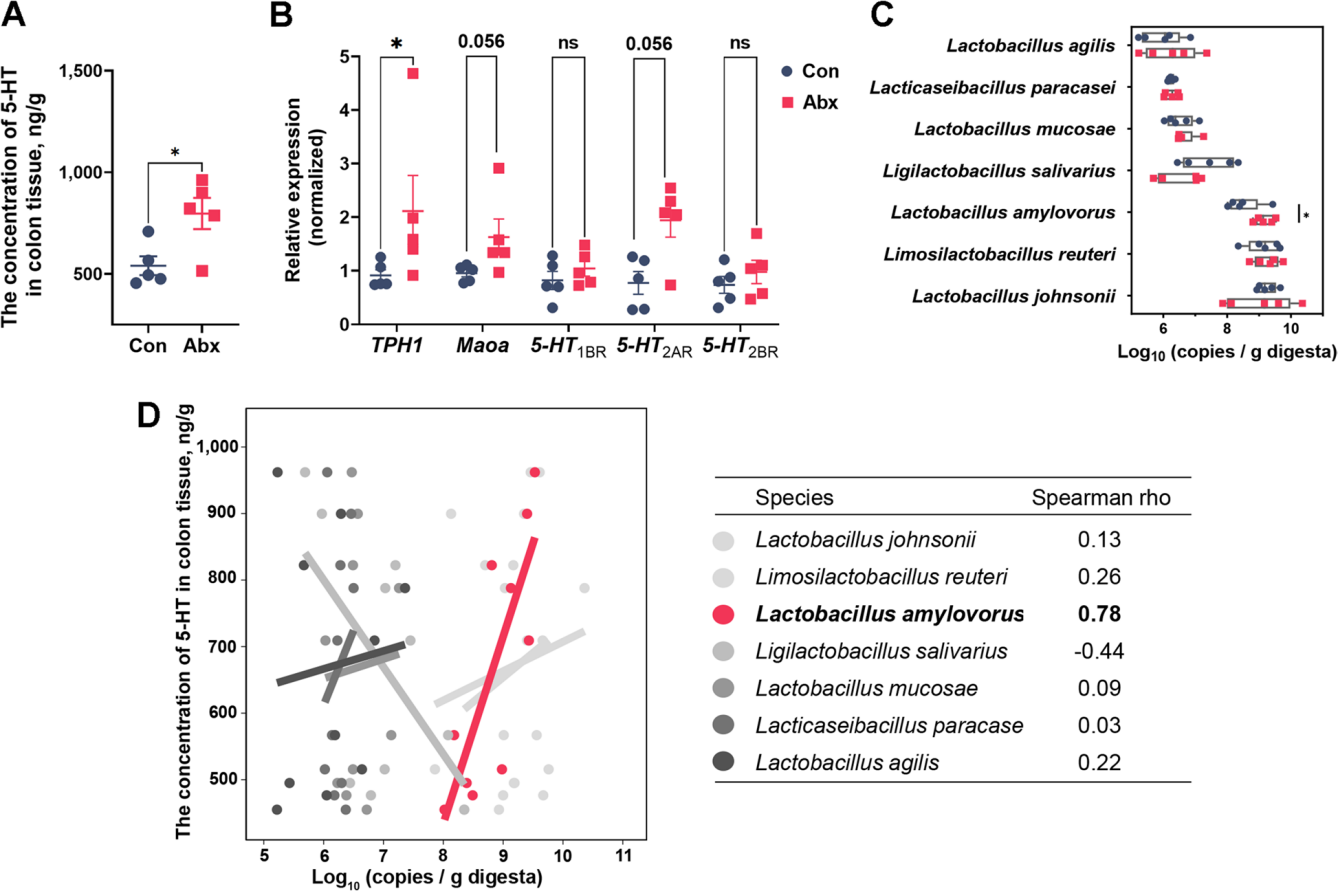
Effects of antibiotic intervention on 5-HT concentration and *Lactobacillus* quantities in the colon of pigs. The effect of antibiotics on 5-HT concentration (**A**), 5-HT pathway gene expression (**B**), and quantities of specific *Lactobacillus* species (**C**). All values are expressed as mean ± SEM. *n* = 5 per group. The Student's *t*-test was performed between two groups while asterisks mean statistically significant difference: ^*^P < 0.05. **D** The correlation analysis between 5-HT concentration and *Lactobacillus* quantities. The rho values showed the correlation between serotonin and *Lactobacillus amylovorus* based on Spearman’s correlation. TPH1: Tryptophan hydroxylase 1; Maoa: Monoamine oxidase A; 5-HT_1B_: 5-Hydroxytryptamine receptor 1B; 5-HT_2AR_: 5-Hydroxytryptamine receptor 2A; 5-HT_2BR_: 5-Hydroxytryptamine receptor 2B

The effects of antibiotics on the 5-HT synthesis and bacterial quantities in the colon are shown in Fig. 1. The antibiotics significantly increased the concentration of 5-HT (Fig. 1A) in the colon tissue (*P* < 0.05). At the level of gene expression (Fig. 1B), the antibiotics up-regulated the gene expression of *TPH1* (*P* < 0.05), which encoded the rate-limiting enzyme for the synthesis of 5-HT. Among genes encoding 5-HT receptor and metabolic enzymes, antibiotics tended to up-regulate monoamine oxidase A (*Maoa*) and *5-HT*_2AR_ expression (0.05 < *P* < 0.1) while not changing the relative expression of *5-HT*_1BR_ and *5-HT*_2BR_.

The effect of antibiotics on colonic dominant microbiota was determined by the quantitative real-time qPCR. The antibiotics did not affect the total bacteria quantities in the colonic digesta (Fig. S3). At the genus level, the quantities of *Clostridium* cluster XIVa, *Roseburia,* and *Ruminococcus* were significantly decreased in the Abx group (*P* < 0.05) while the quantities of *Lactobacillus* tended to be increased (0.05 < *P* < 0.1). At the species level (Fig. 1C), antibiotics significantly increased the quantity of *L. amylovorus* (*P* < 0.05). There was no difference in the quantities of *L. johnsonii*, *L. reuteri*, *L. salivarius*, *L. mucosae*, *L. paracasei,* and *L. agilis* between groups.

To assess the potential relationship between *Lactobacillus* species and 5-HT, the correlation between *Lactobacillus* species quantities and 5-HT concentration was analyzed. The quantity of *L. amylovorus* showed a strong positive correlation with 5-HT concentration (Spearman rho = 0.78) and the quantity of *L. reuteri* showed the weak positive correlation (Spearman rho = 0.26) while no correlations were observed for other *Lactobacillus* species (Fig. 1D). These results suggested that the increased *L. amylovorus,* which was enriched by antibiotics, may affect the 5-HT synthesis in the colon.

### Bacterial isolation and identification

**Fig. 2:**
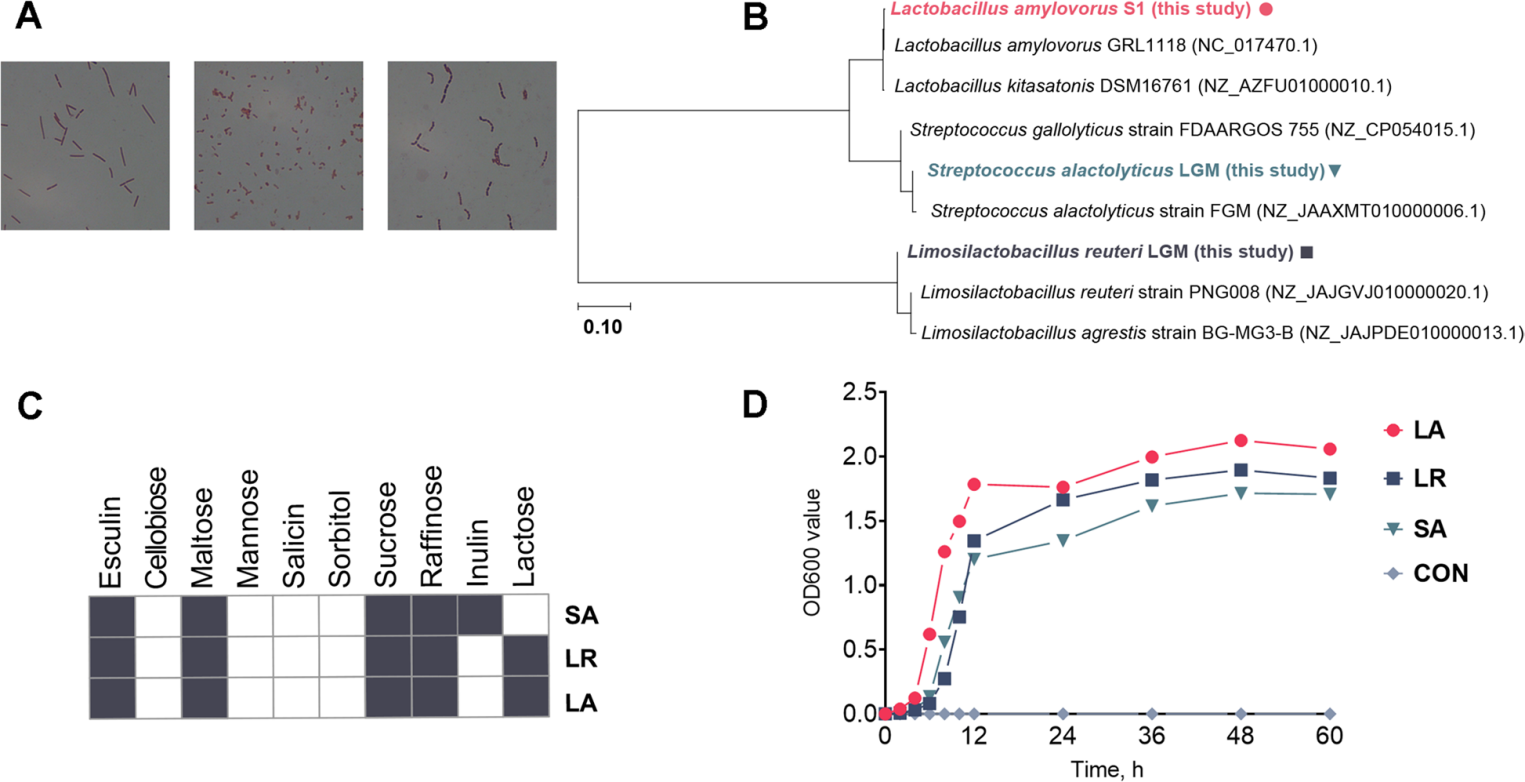
Physiological and biochemical characteristics of *L. amylovorus* S1, *L. reuteri* LGM, and *S. alactolyticus* LGM. **A** The Gram-staining morphology. **B** The phylogenetic tree based on 16S rRNA sequence. **C** The bacterial ability to use different sugars. Dark color represents the ability to utilize while white color represents the inability to utilize specific sugars. **D** Bacterial growth curves from 0 to 60 h

A total of 11 bacterial strains from the pig colon (Table S4), including *L. reuteri* LGM (LR) and *S. alactolyticus* LGM (SA) [20], were isolated. *L. amylovorus* (LA) was also included in the measurement. The Gram-staining results showed that LA with short rods and LR with long rods were two types of Gram-positive *Lactobacillus* with different lengths, and SA was a type of Gram-positive *Streptococcus* (Fig. 2A). Based on the 16S rRNA gene sequence of three strains and those available in the NCBI database, we constructed a phylogenetic tree (Fig. 2B). The biochemical identification showed that both LA and LR could utilize the esculin, maltose, sucrose, raffinose, and lactose (Fig. 2C). Among these sugars, the SA could not utilize the lactose but had the ability to utilize the inulin (Fig. 2C). In the same MRS medium in vitro, the growth rate of LA was significantly higher than the other two strains, while the growth rate of SA was the slowest (Fig. 2D).

### Effects of bacterial supernatant on 5-HT production in vitro

**Fig. 3:**
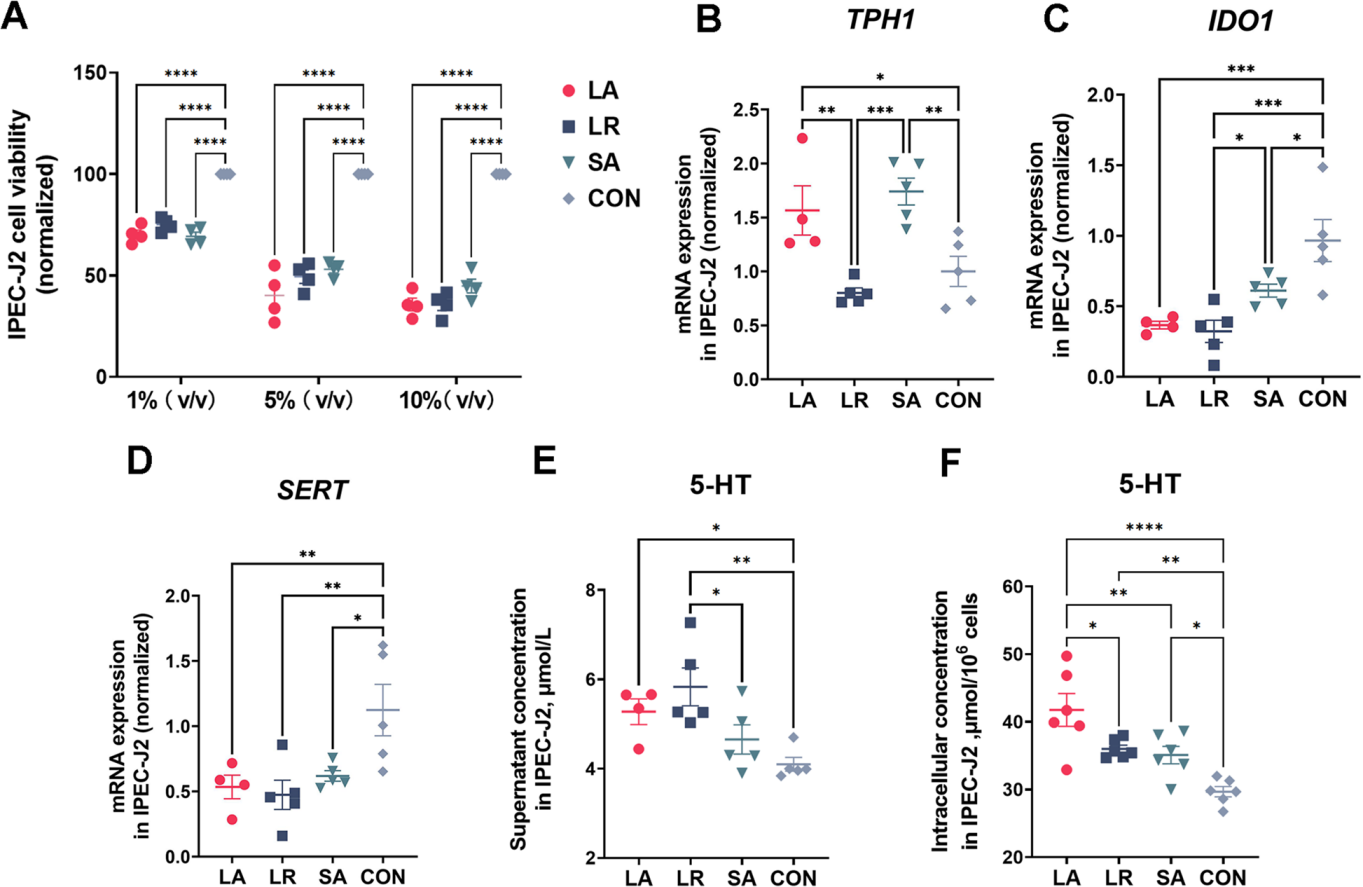
The bacterial supernatants promote 5-HT synthesis in IPEC-J2 cells. **A** The effect of 1%, 5%, and 10% bacterial supernatant on cell viability. The effect of 10% bacterial supernatant on *TPH1* (**B**), *IDO1* (**C**), and *SERT* (**D**) mRNA expression. The effect of 10% bacterial supernatant on cell supernatant (**E**) and intracellular (**F**) 5-HT concentration. All values are mean ± SEM. *n* = 4–6 per group. The one-way ANOVA and multiple comparisons in Fisher’s LSD test were performed while asterisks mean statistically significant difference: ^*^P < 0.05, ^**^P < 0.01, ^***^P < 0.001, ^****^P < 0.0001. TPH1: Tryptophan hydroxylase 1; IDO1: Indoleamine 2,3-dioxygenase 1; SERT: Serotonin reuptake transporter

To verify whether these strains could effectively regulate 5-HT synthesis in vitro, we prepared bacterial supernatants growing at the same OD value. First, we selected porcine IPEC-J2 cell lines and treated them with 1%, 5%, and 10% bacterial culture supernatant, respectively. The cell viability results showed that all three concentrations significantly inhibited (*P* < 0.05) IPEC-J2 cell viability (Fig. 3A), and the 10% bacterial culture supernatant was used for the subsequent experiment. Compared to CON and LR group, the supernatants of LA and SA significantly increased (*P* < 0.05) the gene expression of *TPH1* (Fig. 3B). All the supernatants of three strains down-regulated (*P* < 0.05) the *IDO1* and *SERT* gene expression (Fig. 3C and D). Meanwhile, LR supernatant inhibited *IDO1* expression more significantly than SA (*P* < 0.05). Both LA and LR significantly increased (*P* < 0.05, Fig. 3E) the 5-HT concentration in the cell supernatant while all three strains significantly increased (*P* < 0.05, Fig. 3F) the intracellular 5-HT concentration in the cell. Additionally, the concentration of 5-HT in LR group was significantly higher than that in SA group (*P* < 0.05, Fig. 3E). The intracellular 5-HT concentration in LA group was significantly higher than that in LR and SA groups (*P* < 0.05, Fig. 3F).

**Fig. 4:**
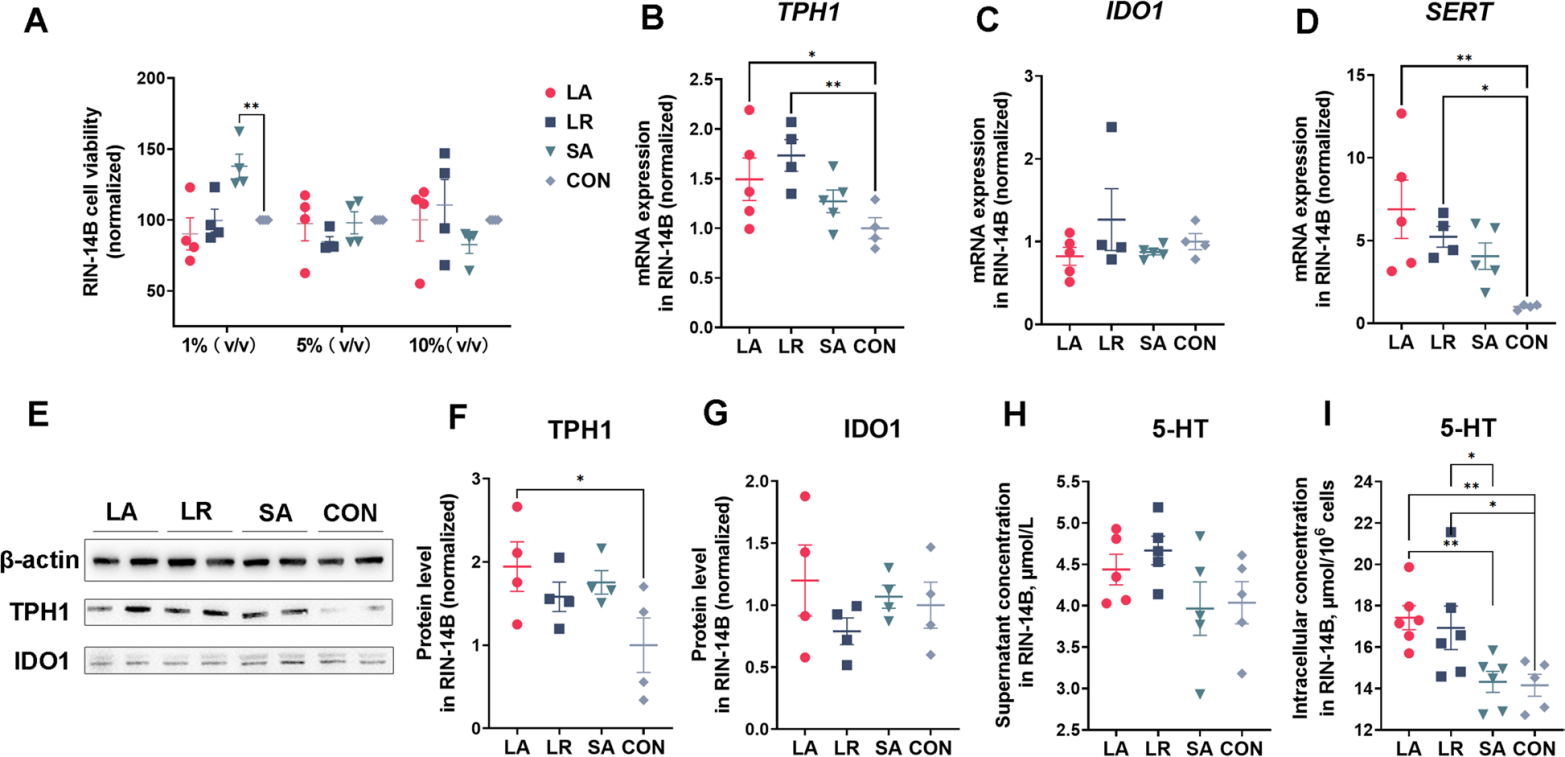
The bacterial supernatants promote 5-HT synthesis in RIN-14B cells. **A** The effect of 1%, 5%, and 10% bacterial supernatant on cell viability. The effect of 10% bacterial supernatant on* TPH1* (**B**), *IDO1* (**C**), and *SERT* (**D**) mRNA expression. **E** The Western blot strip of β-actin, TPH1, and IDO1. The effect of 10% bacterial supernatant on TPH1 (**F**) and IDO1 (**G**) protein level. The effect of 10% bacterial supernatant on cell supernatant (**H**) and intracellular (**I**) 5-HT concentration. All values are mean ± SEM. *n* = 4–6 per group. The one-way ANOVA and multiple comparisons in Fisher’s LSD test were performed while asterisks mean statistically significant difference: ^*^P < 0.05, ^**^P < 0.01. TPH1: Tryptophan hydroxylase 1; IDO1: Indoleamine 2,3-dioxygenase 1; SERT: Serotonin reuptake transporter

RIN-14B cells, which was the enterochromaffin homolog cell, were further used to evaluate the bacterial regulation of serotonin production. Inconsistent with IPEC-J2 cell results, 1%, 5%, and 10% bacterial culture supernatants did not affect cell viability in the RIN-14B cells (Fig. 4A). At the gene level, the LA and LR supernatants significantly increased (*P* < 0.05) the *TPH1* and *SERT* gene expression (Fig. 4B and D) while did not affect the *IDO1* (Fig. 4C). At the protein level, only LA supernatant significantly up-regulated the TPH1 protein levels with no effect on the IDO1 (Fig. 4E, F and G). All the bacterial supernatants did not affect the 5-HT concentration in the cell supernatant while both LA and LR significantly increased (*P* < 0.05) the intracellular 5-HT concentration in the RIN-14B compared to CON and SA groups (Fig. 4H and I).

### Computational prediction and measurement of metabolites in the bacterial supernatant

**Fig. 5:**
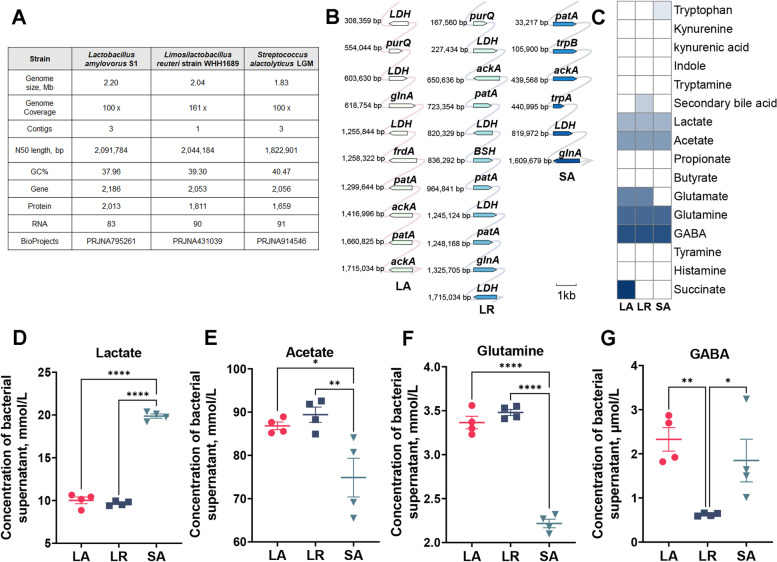
Computational prediction and ability of metabolite production in *L. amylovorus* S1, *L. reuteri* LGM, and *S. alactolyticus* LGM. **A** Comparison of whole genome information of three strains. **B** The key genes encoding bacterial metabolic enzymes and their localization in the chromosome. **C** Prediction of metabolite production in three strains. The lactate (**D**), acetate (**E**), glutamine (**F**), and GABA (**G**) concentrations in bacterial supernatant (*n* = 3). All values are mean ± SEM. ^*^P < 0.05, ^**^P < 0.01, ^***^P < 0.001, ^****^P < 0.0001. LDH: *L*-lactate dehydrogenase; ackA: Acetate kinase; glnA: Glutamine synthetase; patA: Aminotransferase; purQ: Glutaminase; frdA: Fumarate reductase; BSH: Choloylglycine hydrolase; trpA/trpB: Tryptophan synthase

To find out which metabolites in the bacterial supernatant may effectively promote 5-HT synthesis, we predicted the ability of bacteria to produce metabolites using the bacterial whole genome. We obtained the whole genome sequences of LA in our previous study [25] and downloaded the whole genome sequence of the *L. reuteri* strain WHH1689, which showed the highest similarity with *L. reuteri* LGM in 16S sequence among 33 existing *L. reuteri* genomes from NCBI (Fig. S2). The complete genome of SA, sequenced in this study, consists of a circular chromosome (1,822,901 bp), and 2 circular plasmids (4,732 bp and 4,565 bp, respectively). The whole genome of LA, LR, and SA contained 2.20 Mb, 2.04 Mb, and 1.83 Mb data, respectively (Fig. 5A). The genome of LA, LR, and SA contained 2,013, 1,811, and 1,659 protein-coding sequences, respectively. Among the genes that encode proteins, we focused on those commonly produced by microbes in the gut (Fig. 5B and C). The LA, LR, and SA can produce lactate, acetate, glutamine, and GABA under the function of *L*-lactate dehydrogenase (LDH), acetate kinase (ackA), glutamine synthetase (glnA) and putrescine aminotransferase (patA). Both the LA and LR can produce glutamate by glutaminase (purQ). Only LA can produce the succinate through fumarate reductase (frdA) while LR can produce the secondary bile acid by choloylglycine hydrolase (BSH) and SA can synthesize the tryptophan by tryptophan synthase (trpA/trpB).

To test whether the metabolite levels were consistent with the gene function prediction, we examined the concentrations of metabolites that were present in the three bacterial supernatants with the same OD value. The concentration of lactate was around 10 mmol/L in the LA and LR while 20 mmol/L in the SA (Fig. 5D). The lactate in LA and LR supernatants were significantly lower than in SA (*P* < 0.05). The concentration of acetate was around 80 mmol/L in the LA and LR, significantly higher than in SA (*P* < 0.05, Fig. 5E). The concentration of glutamine was more than 3 mmol/L in the LA and LR while around 2 mmol/L in the SA supernatants (Fig. 5F). The glutamine in LA and LR supernatants were significantly higher than in SA (*P* < 0.05). The concentration of GABA was around 2 μmol/L in the LA and SA while below 1 μmol/L in the LR supernatants (Fig. 5G). The GABA in LA and SA supernatants were significantly higher than in LR (*P* < 0.05).

### Effect of bacterial metabolites on 5-HT synthesis

To figure out which metabolite in the bacterial supernatant promotes the synthesis of 5-HT in cells, we treated IPEC-J2 cells with 10 metabolites. Among these metabolites, lactate, acetate, glutamine, GABA, and succinate could be detected in the supernatant of LA, LR, and SA, while propionate, butyrate, deoxycholate, kynurenine, and kynurenic acid may not be produced by these strains. The concentrations of lactate (1 mmol/L), acetate (10 mmol/L), glutamine (0.3 mmol/L), and GABA (1 μmol/L) were configured according to the concentration in the 10% bacterial supernatant while succinate (1 mmol/L), propionate (200 μmol/L), butyrate (200 μmol/L), deoxycholate (25 μmol/L), kynurenine (200 μmol/L) and kynurenic acid (100 μmol/L) concentrations were based on previous studies.

**Fig. 6:**
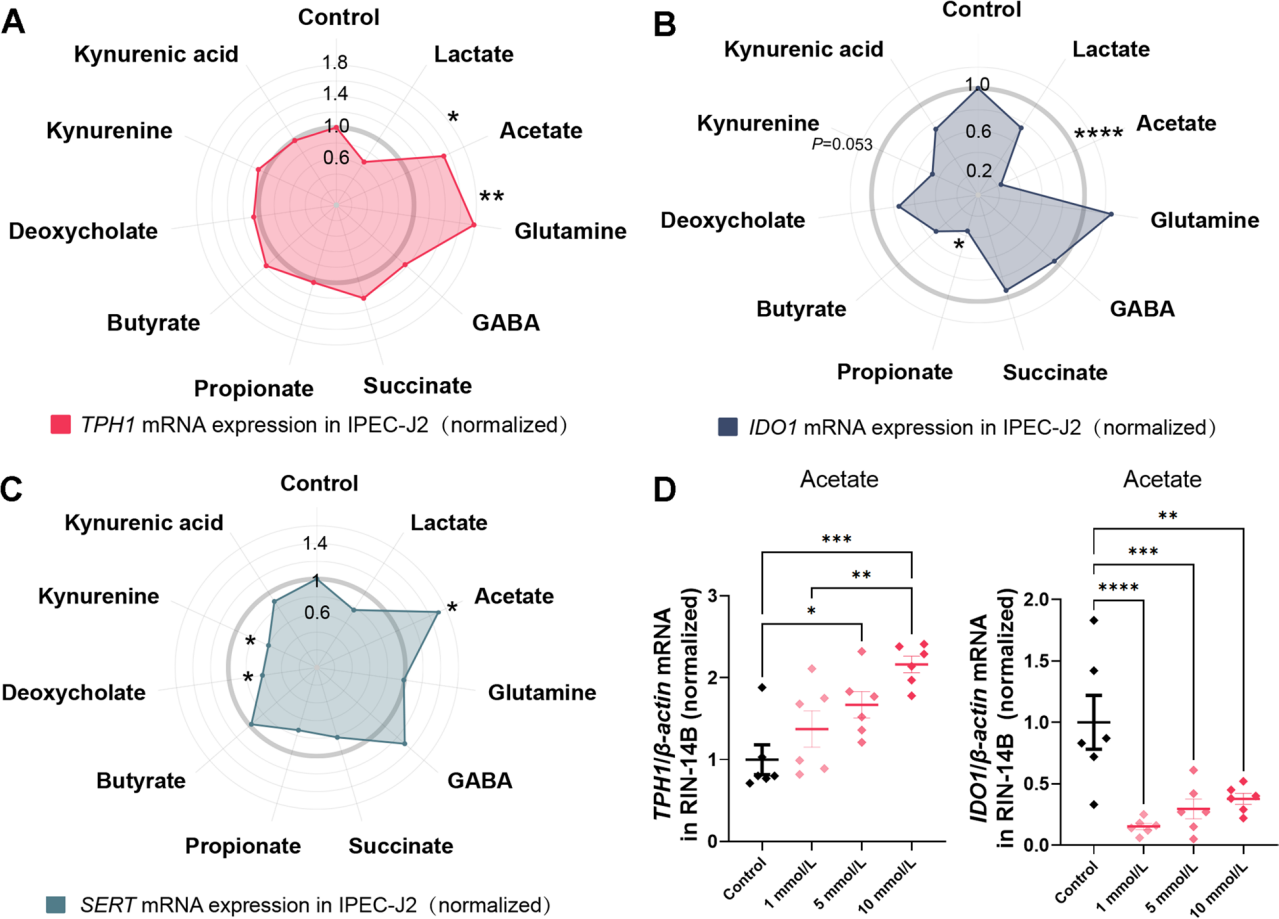
The bacterial metabolites promote 5-HT synthesis in IPEC-J2 and RIN-14B cells. The effect of bacterial metabolites on *TPH1* (**A**), *IDO1* (**B**), and *SERT* (**C**) mRNA expression in IPEC-J2 cells. The effect of acetate on *TPH1* (**D**) and *IDO1* (**E**) mRNA expression in RIN-14B cells. All values are mean ± SEM. *n* = 6 per group. The Student's *t*-test was performed between two groups while asterisks mean statistically significant difference: ^*^P < 0.05, ^**^P < 0.01, ^***^P < 0.001, ^****^P < 0.0001. TPH1: Tryptophan hydroxylase 1; IDO1: Indoleamine 2,3-dioxygenase 1; SERT: Serotonin reuptake transporter

The results showed that both acetate and glutamine could significantly up-regulate (*P* < 0.05) the *TPH1* gene expression in the IPEC-J2 cells (Fig. 6A). Both acetate and propionate could significantly down-regulate (*P* < 0.05) the *IDO1* gene expression and kynurenine had the tendency (*P* = 0.053) for decreasing *IDO1* expression (Fig. 6B). In addition, acetate could significantly up-regulate (*P* < 0.05) the *SERT* gene expression while deoxycholate and kynurenine significantly (*P* < 0.05) down-regulated *SERT* in the IPEC-J2 cells (Fig. 6C).

To further verify the effect of acetate on *TPH1*, we repeated the experiment on RIN-14B cells. RIN-14B cells were treated with 1, 5, and 10 mmol/L acetate, respectively. The results showed that both 5 mmol/L and 10 mmol/L acetate could significantly up-regulate (*P* < 0.05, Fig. 6D) the *TPH1* gene expression while 1 mmol/L acetate could not. In addition, 1, 5, and 10 mmol/L acetate all significantly down-regulated (*P* < 0.05, Fig. 6D) the *IDO1*gene expression.

### The effect of acetate on histone acetylation and its receptor in cells

**Fig. 7:**
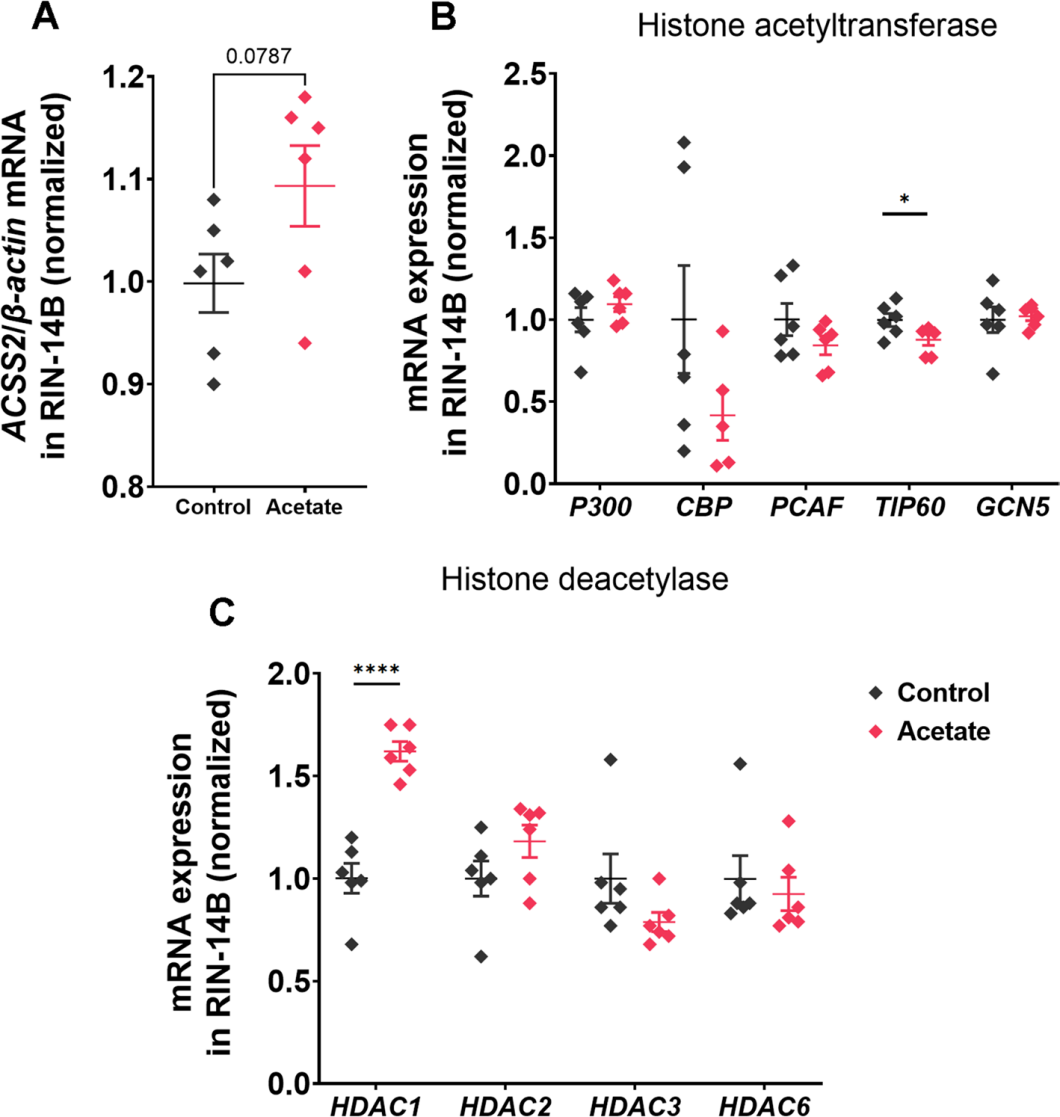
The effect of acetate on histone acetylation in RIN-14B cells. **A** The effect of acetate on *ACSS2* mRNA expression. The effect of acetate on histone acetyltransferase (**B**) and histone deacetylase (**C**) mRNA expression. All values are mean ± SEM. *n* = 6 per group. The Student's *t*-test was performed between two groups while asterisks mean statistically significant difference: ^*^P < 0.05, ****P < 0.0001. ACSS2: Acetyl-CoA synthetase 2; HDAC: Histone deacetylation

To further examine how acetate may act on cells, we focused on the effects on histone acetylation, deacetylase and acetate receptors. First, we investigated whether acetate affected intracellular histone acetylation to regulate *TPH1* by affecting the synthesis of acetyl-CoA in the RIN-14B cells. The results showed that acetate had the tendency (0.05 < *P* < 0.1) for upregulating acetyl-CoA synthetase 2 (*ACSS2*) gene expression (Fig. 7A). Among genes related to histone acetylation, acetate significantly decreased (*P* < 0.05) the gene expression of *TIP60* but did not affect *P300*, *CBP*, *PCAF*, and *GCN5* (Fig. 7B). Concerning genes involved in histone deacetylation (HDAC), that acetate significantly increased (*P* < 0.05) the gene expression of *HDAC1* but did not affect *HDAC2*, *HDAC3*, and *HDAC6* (Fig. 7C). These results suggested that acetate may decrease the level of intracellular acetylation and increase the level of deacetylation.

**Fig. 8:**
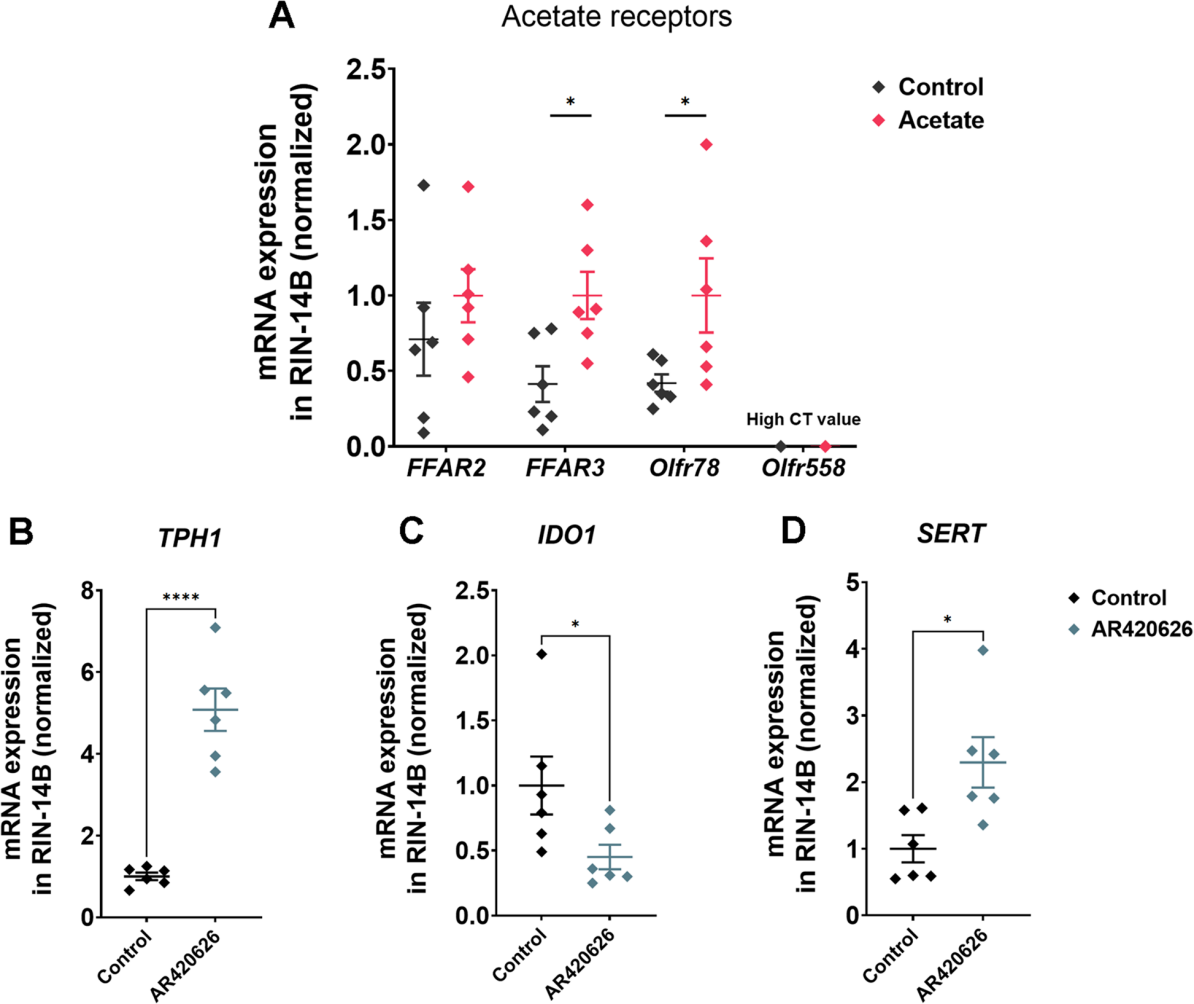
The effect of acetate on its receptors in RIN-14B cells. **A** The effect of acetate on its receptors mRNA expression. The effect of AR420626 on *TPH1* (**B**), *IDO1* (**C**) and *SERT* (**D**) mRNA expression. All values are mean ± SEM. *n* = 6 per group. The Student's *t*-test was performed between two groups while asterisks mean statistically significant difference: ^*^P < 0.05, ^****^P < 0.0001. ACSS2: Acetyl-CoA synthetase 2; HDAC: Histone deacetylation. FFAR2: Free fatty acid receptor 2; FFAR3: Free fatty acid receptor 3; Olfr78: Olfactory receptor 78; Olfr558: Olfactory receptor 558; TPH1: Tryptophan hydroxylase 1; IDO1: Indoleamine 2,3-dioxygenase 1; SERT: Serotonin reuptake transporter

Next, we focused on the effect of acetate on the mRNA expression of its receptor. Primarily, we observed the effect of 10 mmol/L acetate on different receptors including the free fatty acid receptor 2 (FFAR2), free fatty acid receptor 3 (FFAR3), olfactory receptor 78 (Olfr78) and olfactory receptor 558 (Olfr558). The results showed that 10 mmol/L acetate did not affect the gene expression of *FFAR2* (Fig. 8A). Conversely, 10 mmol/L acetate upregulated the *FFAR3* and *Olfr78* mRNA expression. However, we failed to detect the *Olfr558* expression in RIN-14B cells (Fig. 8A). FFAR2 and FFAR3 are the main receptors of acetate. Therefore, we further studied the effect of antagonizing or activating these two types of receptors on 5-HT synthesis. The results showed that adding 0.1 μmol/L GLPG0974, the antagonist of FFAR2, did not block the upregulation of *TPH1* by acetate (Fig. S4A). Meanwhile, the expression of *IDO1* and *SERT* were not affected by the addition of GLPG0974 (Fig. S4B and C). Importantly, we found that 10 μmol/L AR420626, the agonist for FFAR3, significantly increased *TPH1* mRNA expression to the same degree as acetate (Fig. 8B). Additionally, the *IDO1* expression was inhibited and the *SERT* expression was upregulated (Fig. 8C and D).

## Discussion

In the present study, we found that antibiotics significantly elevated the 5-HT levels and TPH1 expression as well as increased *Lactobacillus amylovorus* quantity in the colon of piglets. The positive correlation between *Lactobacillus amylovorus* and 5-HT was further validated using a series of cell culture studies in vitro. Mechanistically, acetate in the supernatant of *Lactobacillus amylovorus* S1 increased the TPH1 expression both in IPEC-J2 and RIN-14B cells, the action of which was involved in the acetate receptor FFAR3. These findings support the microbial regulation on intestinal 5-HT production in piglets.

The present study identified a close correlation between intestinal microorganisms and intestinal TPH1 expression, the rate-limiting enzyme for mucosal 5-HT synthesis. Previous studies have revealed that antibiotic intervention significantly affected the richness and diversity of intestinal microbes in mice, accompanied by a significant decrease in *TPH1* expression [11]. Colonizing germ-free mice with human microbiota significantly increased the colonic *TPH1* expression [17]. However, the interaction between gut microbiota and serotonin production in pigs was unclear. Interestingly, employing ileum-cannulated pigs after the antibiotic intervention, we found a significant increase in colonic *TPH1* expression and 5-HT levels, indicating the potential involvement of gut microbiota in  serotonin production. To figure out which microbes might be closely related to an increase in 5-HT, we quantified the amount of representative bacteria in the colon and demonstrated that only *Lactobacillus* showed a consistent direction of change as 5-HT .

The *Lactobacillus* exhibited prominent antibiotic resistance in the gut. Similarly, previous study has shown that *L. amylovorus* strains from domestic pigs were predominantly resistant to ampicillin [27], which was part of the antibiotic recipe used in the present study. Interestingly, genome anlaysis showed that the *L. amylovorus* S1 harbors the *tetW* gene that confers resistance to tetracycline, which may contribute to the increase of *Lactobacillus* after antibiotic intervention. The increase of 5-HT in vivo may be due to the upregulated expression of key enzymes for serotonin production, namely the tryptophan hydroxylase 1. Antibiotics may affect the microbial utilization of tryptophan [20] and thus increase tryptophan availability for mucosal production of serotonin, which still needs more investigation.

Results of the current study suggest that *L. amylovorus* S1 isolated from pig gut could signficantly upregulate TPH1 expression and 5-HT synthesis both in IPEC-J2 and RIN-14B cells. It has been documented that *Lactobacillus* species such as *Lactobacillus plantarum* and *Lactobacillus reuteri* can not only promote 5-HT secretion in the human and mice [28, 29], but also regulate gut motility and attenuate stress and depression through microbiota-gut-brain axis [30, 31]. Additionally, we further elucidated that there was a competitive relationship between kynurenine and the 5-HT pathway. Tryptophan in the gut can be metabolized by the host into two main pathways: the 5-HT synthetic pathway via TPH1 and the kynurenine degradation pathway via IDO1 [32]. We observed that *L. amylovorus* S1 and its metabolite acetate could up-regulate the gene expression of *TPH1* while down-regulate the *IDO1* in vitro, which was concordant with other studies showing reverse direction of change between the level of 5-HT and kynurenine in vivo [33–35]. 

Another interesting finding can be embodied in the different trends of *SERT* relative mRNA expression in IPEC-J2 and RIN-14B cells. Previous studies have shown that gut microbes may play an essential role in regulating*  SERT* expression [5, 36]. We noticed that *Lactobacillus* supernatant significantly inhibited the expression of *SERT* in IPEC-J2 cells, but upregulated *SERT* in RIN-14B cells. SERT is a type of membrane protein, which transported the extracellular 5-HT back to the intracellular environment to ensure its recycling [37]. Impaired uptake of 5-HT due to impeditive *SERT* expression in the gut can lead to increased 5-HT available in the body [38]. Abnormal *SERT* expression in patients may be associated with irritable bowel syndrome (IBS) [39]. Interestingly, we found that bacterial supernatant promoted 5-HT synthesis both in IPEC-J2 and RIN-14B cells, but had the opposite effect on supernatant 5-HT. Combined with inverse *SERT* expression in two cells, we speculated that the lower *SERT* expression in IPEC-J2 cells may hinder the released 5-HT in supernatant back into intracellular space for recycling. On the contrary, an increase in *SERT* expression in RIN-14B cells may accelerate the released 5-HT back into the intracellular space, resulting in similar 5-HT levels between groups. However, the reasons for the discrepancy across cell types remained unknown in this study, which may be consequences of different origins and physiological characteristics in IPEC-J2 and RIN-14B cells [16, 40, 41].

Microbial metabolites may mediate the impact of microbes on *TPH1* expression. Based on current findings, we demonstrated that acetate and glutamine could up-regulate the gene expression of *TPH1* in RIN-14B cells. The acetate attracted our attention for two main reasons. Firstly, only acetate could increase *TPH1* expression while inhibiting *IDO1* expression, which was consistent with our previous results using bacterial supernatants. Secondly, glutamine was originally present in the 1640 medium for RIN-14B cell growth, and we did not exclude this factor at the beginning of the experiment. Notably, we also revealed that kynurenine, another metabolite in the tryptophan metabolic pathway, had the tendency for decreasing *IDO1* gene expression and significantly inhibited the *SERT* gene expression, suggesting that more kynurenine may hamper the entry of tryptophan into the kynurenine pathway. These results were also in line with our deduction that there was a competitive relationship between kynurenine and the 5-HT pathway. 

Previous studies have documented that histone deacetylation may be involved in the regulation of *TPH1* expression. Inhibition of histone deacetylation expression by *HDAC1* and *HDAC3* promoted *TPH1* expression and endogenous serotonin synthesis in rat [42]. Interestingly, acetate could be used to synthesize acetyl-CoA under the action of ACSS2 and participate in the regulation of histone deacetylation [43, 44]. The findings raised our interest that whether acetate-induced higher *TPH1 *expression was associated with inhibited histone deacetylation. However, we found that acetate increased *TPH1* expression and simultaneously up-regulated *HDAC1* gene expression, suggesting a possible increase in histone deacetylation levels. 

Gut microbiota is known to affect gut-brain axis activity via metabolites including acetate [45]. Short-chain fatty acids such as acetate in the gut can act on a wide variety of receptors. FFAR2 and FFAR3 belong to G protein-coupled receptors recognizing acetate [46]. Recent studies reported that acetate can act on mouse Olfr78 and Olfr558, but the specific regulatory mechanism remains unclear [47]. In the present study, we found that acetate increased the expression of *FFAR3* and *Olfr78* with no significant effect on *FFAR2*. GLPG0974 and AR420626 are commonly used antagonists and agonists, respectively, to study  the action of FFAR2 and FFAR3 [48, 49]. In order to verify the role of acetate receptors , we used GLPG0974 and AR420626 in the study. The results showed that inhibiting FFAR2 receptors did not affect *TPH1* expression, but activation of FFAR3 receptors significantly increased *TPH1* expression. Overall, the finding implicates the involvement of FFAR3 in *TPH1* expression, but it is unknown whether the regulation is FFAR3-dependent, which remains to be solved in future studies.

There are several limitations in this study. Different strains of bacteria within the same species may indeed have different characteristics in their physiological and biochemical properties. Whether all strains of *L. amylovorus* are able to influence serotonin production in the gut remains unknown and is a meaningful question to pursue in future study. Additionally, it is important to acknowledge that only limited animals were used in the study. Future investigation with more replicates will be helpful to explore how *L. amylovorus* and acetate regulate 5-HT biosynthesis in vivo.

## Conclusion

In this study, we found that antibiotic intervention significantly affected the 5-HT synthesis and bacteria quantities in the colon of piglets. Correlation analysis revealed that *L. amylovorus* was positively correlated with the 5-HT concentration. Cell culture studies further confirm the positive regulation of *L. amylovorus* on serotonin production. The microbial metabolite acetate could up-regulate the *TPH1* expression both in the IPEC-J2 and RIN-14B cells. Importantly, we found that acetate may act on FFAR3 to promote *TPH1* expression. Overall, inspired by the in vivo findings in piglets, we verified that the strain of *L. amylovorus* and its metabolites could regulate the 5-HT biosynthesis in vitro. These findings reveal the potential microbial regulation on 5-HT production in the pigs and provide evidence of how specific microbes and their metabolites interact with host.

## Supplementary Information

**Additional file 1: Table S1.** The composition and analyzed nutrient contents of experimental diets.** Table S2.** The primer sequences of bacteria. **Table S3.** The primer sequences of genes. **Table S4.** The identity of isolated bacteria.

**Additional file 2: Fig. S1.**  The experimental timeline.

**Additional file 3: Fig. S2.** The 16S rRNA sequence similarity between different *L. reuteri *species.

**Additional file 4: Fig. S3.** The effect of antibiotics on major bacteria quantities. All values are expressed as mean ± SEM. *n *= 5 per group. The Student's *t*-test was performed between two groups while asterisks mean statistically significant difference: ^*^P < 0.05.

**Additional file 5: Fig. S4.** The effect of GLPG0974 and acetate on 5-HT related mRNA expression. (A) *TPH1*, (B) *IDO1* and (C) *SERT* mRNA expression. All values are mean ± SEM. *n* = 6 per group. The Student's *t*-test was performed between two groups while asterisks mean statistically significant difference: ^*^P < 0.05, ^****^P < 0.0001. TPH1: Tryptophan hydroxylase 1; IDO1: Indoleamine 2,3-dioxygenase 1; SERT: Serotonin reuptake transporter.

## Data Availability

The 16S rRNA gene sequence of *L. amylovorus* S1, *L. reuteri* LGM, and *S. alactolyticus* LGM have been submitted to NCBI under the accession number MT525371, OQ473051, and OQ473052. The complete sequence of *S. alactolyticus* LGM is available in the NCBI genome database under PRJNA914546.

## Abbreviations

5-HT: 5-Hydroxytryptamine
ackA: Acetate kinase
ACSS2: Acetyl-CoA synthetase 2
BSH: Choloylglycine hydrolase
FFAR2: Free fatty acid receptor 2
FFAR3: Free fatty acid receptor 3
frdA: Fumarate reductase
glnA: Glutamine synthetase
HDAC: Histone deacetylation
IDO1: Indoleamine 2,3-dioxygenase 1
LDH: *L*-lactate dehydrogenase
Maoa: Monoamine oxidase A
Olfr78: Olfactory receptor 78
Olfr558: Olfactory receptor 558
patA: Putrescine aminotransferase
purQ: Glutaminase
SERT: Serotonin reuptake transporter
TPH1: Tryptophan hydroxylase 1
TPH2: Tryptophan hydroxylase 2
trpA / trpB: Tryptophan synthase
