# Supplementary figures and images for: Regulation of serotonin production by specific microbes from piglet gut
Source: J Anim Sci Biotechnol. 2023 Aug 5;14:111. doi: 10.1186/s40104-023-00903-7 (PMC10403853; doi:10.1186/s40104-023-00903-7)

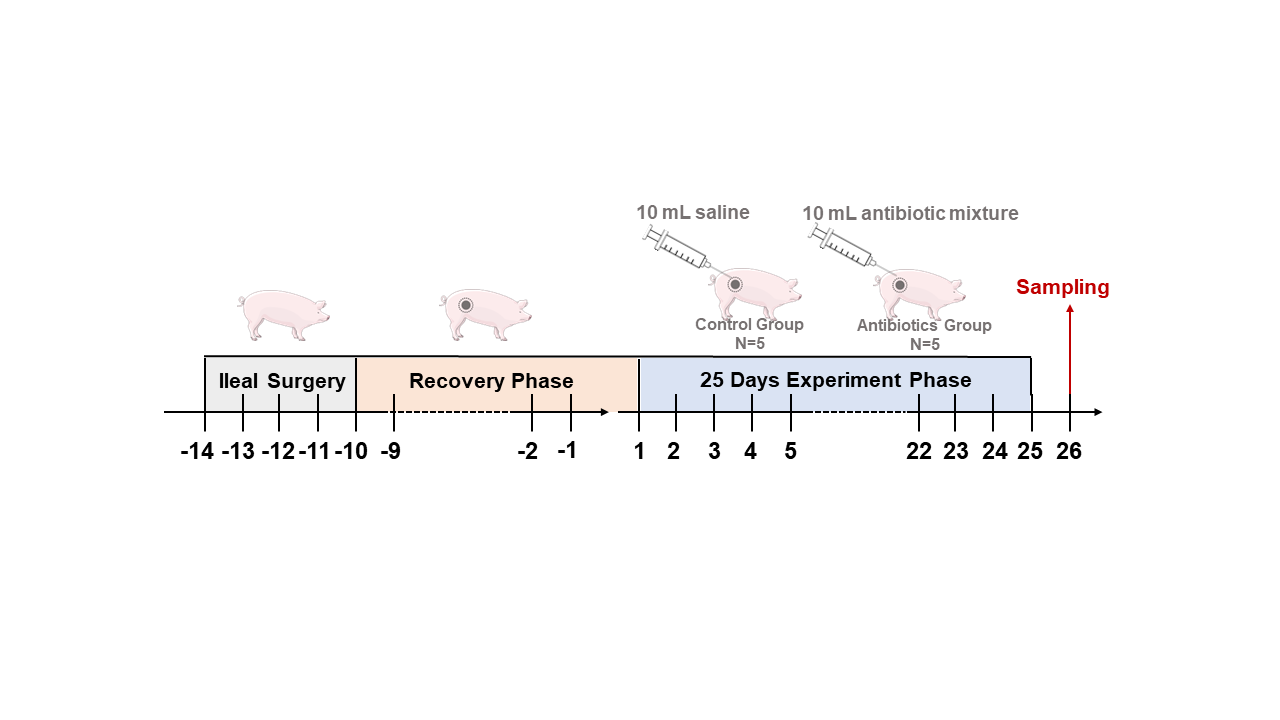

Supplement: Supplementary file 2 — Additional file 2: Fig. S1. The experimental timeline. [file 40104_2023_903_MOESM2_ESM.tif]

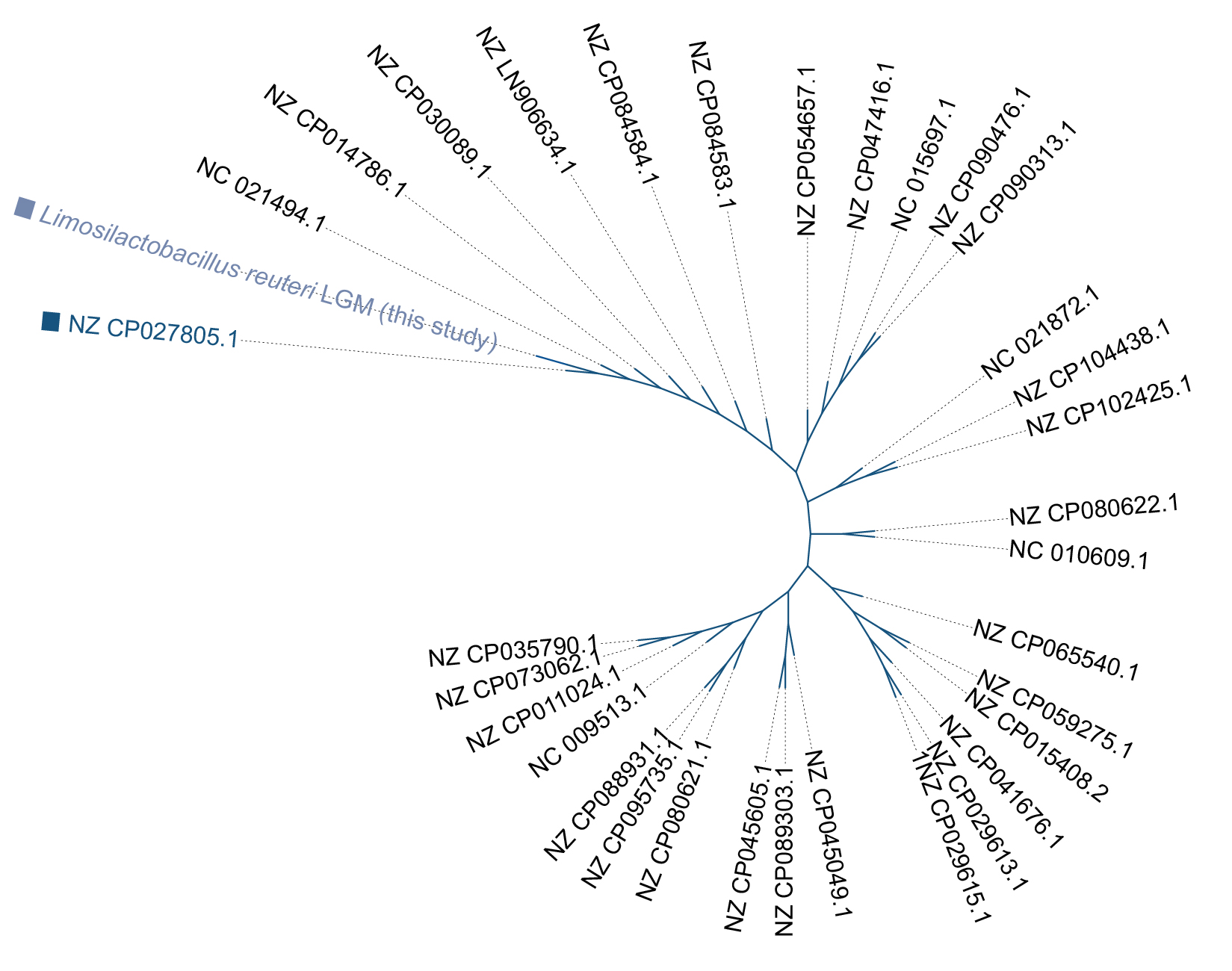

Supplement: Supplementary file 3 — Additional file 3: Fig. S2. The 16S rRNA sequence similarity between different L. reuteri species. [file 40104_2023_903_MOESM3_ESM.jpg]

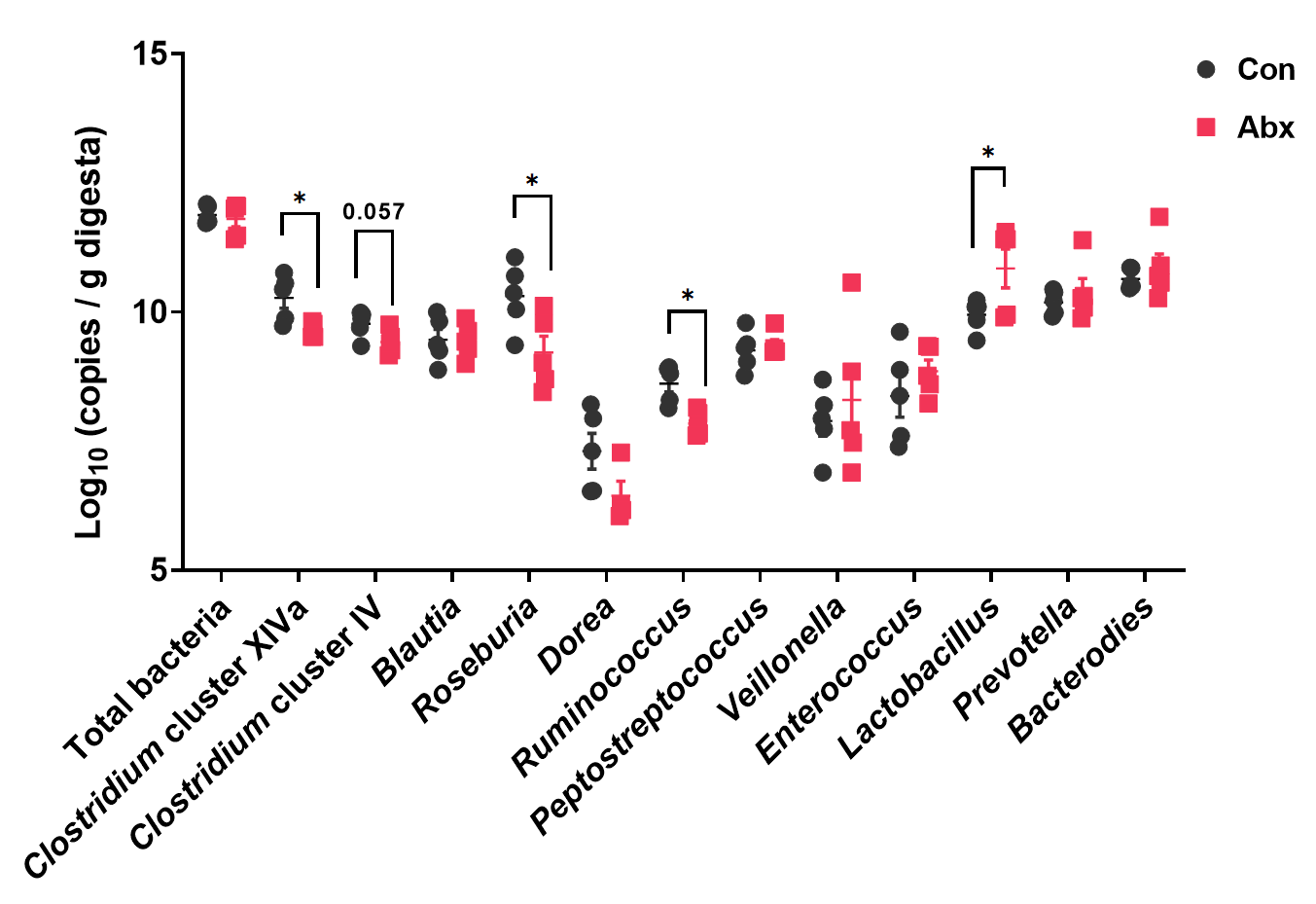

Supplement: Supplementary file 4 — Additional file 4: Fig. S3. The effect of antibiotics on major bacteria quantities. All values are expressed as mean ± SEM. n = 5 per group. The Student's t-test was performed between two groups while asterisks mean statistically significant difference: *P < 0.05. [file 40104_2023_903_MOESM4_ESM.tif]

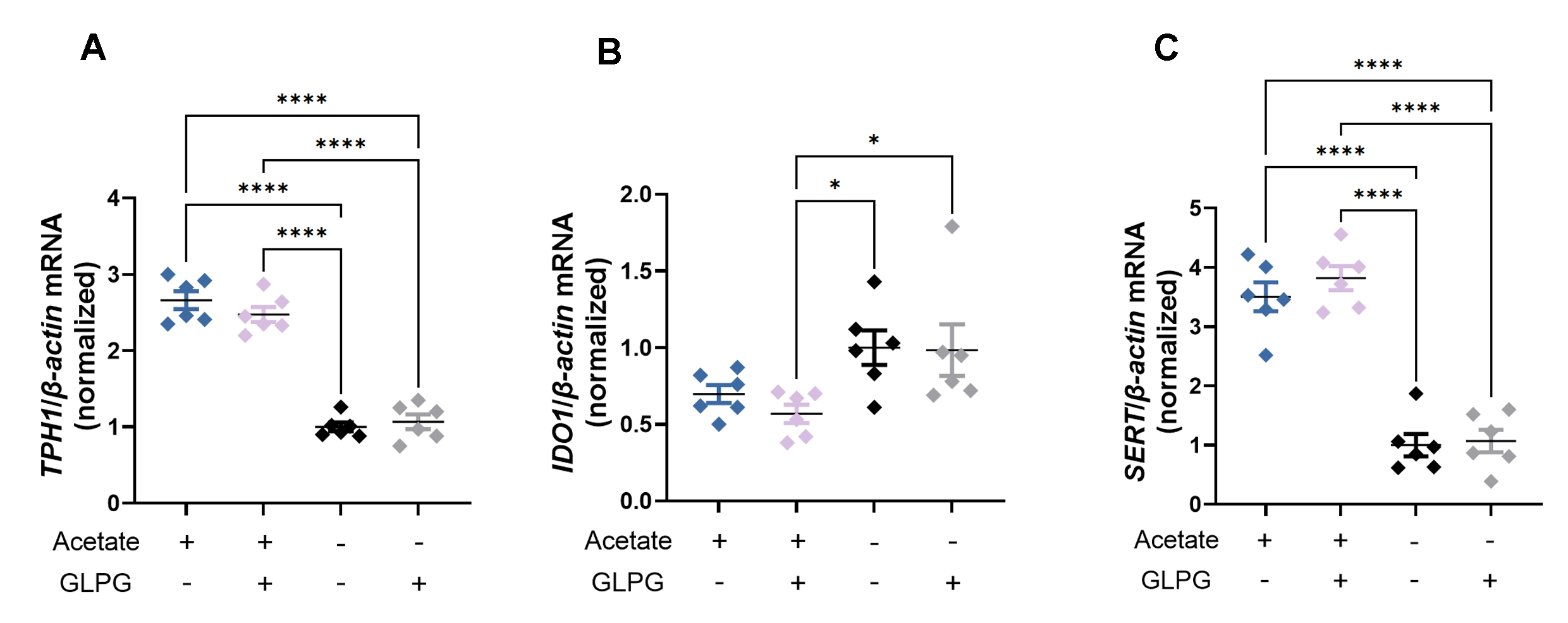

Supplement: Supplementary file 5 — Additional file 5: Fig. S4. The effect of GLPG0974 and acetate on 5-HT related mRNA expression. (A) TPH1, (B) IDO1 and (C) SERT mRNA expression. All values are mean ± SEM. n = 6 per group. The Student's t-test was performed between two groups while asterisks mean statistically significant difference: *P < 0.05, ****P < 0.0001. TPH1: Tryptophan hydroxylase 1; IDO1: Indoleamine 2,3-dioxygenase 1; SERT: Serotonin reuptake transporter. [file 40104_2023_903_MOESM5_ESM.tif]
